# 

**Published:** 1879-07

**Authors:** 


### CONTENTS.

### COMMUNICATIONS.

Address. *By Dr. F. Peabody, President*.................................... 273

Anæsthesia and Anæsthetics. *By Dr. J. J. Caldwell, of Baltimore, Md.* 280

Rip Van Winkle, Dentist. *By W. H. Robinson, Suison, Cal*............ 287

### PROCEEDINGS OF SOCIETIES.

Twentieth Meeting of the Northern Ohio Dental Association.......... 299

### EDITORIAL.

American Dental Association.................................................. 310
Obituary........................................................................ 311
American Society of Microscopists.......................................... 312
Virginia State Dental Association........................................... 313
A Will........................................................................... 313
A New Dental College.......................................................... 314
Wisconsin....................................................................... 315
New Jersey...................................................................... 316

